# Peripheral Autoimmune Regulator Induces Exhaustion of CD4^+^ and CD8^+^ Effector T Cells to Attenuate Autoimmune Diabetes in Non-Obese Diabetic Mice

**DOI:** 10.3389/fimmu.2017.01128

**Published:** 2017-09-15

**Authors:** Divakar Kulshrestha, Li-Tzu Yeh, Ming-Wei Chien, Feng-Cheng Chou, Huey-Kang Sytwu

**Affiliations:** ^1^Molecular and Cell Biology, Taiwan International Graduate Program, Academia Sinica and Graduate Institute of Life Sciences, National Defense Medical Center, Taipei, Taiwan; ^2^Department and Graduate Institute of Microbiology and Immunology, National Defense Medical Center, Taipei, Taiwan

**Keywords:** autoimmune regulator, autoimmune diabetes, dendritic cells, T cell exhaustion, effector T cells, regulatory T cells

## Abstract

Autoimmune regulator (*Aire*) is one of the most crucial genes expressed in the thymus, where it orchestrates the promiscuous expression and presentation of tissue-specific antigens during thymocyte selection. The presence of Aire-expressing cells outside the thymus points toward its plausible extrathymic functions; however, the relative contribution of Aire-expressing cells of hematopoietic origin and their role in the modulation of autoimmune diseases are still obscure. Here, we report that non-obese diabetic mice with transgenic Aire expression under the control of the CD11c (integrin alpha X) promoter were significantly protected from autoimmune diabetes compared with their non-transgenic littermates. The protective effect of *Aire* transgene was mediated primarily by an increase in the “exhausted” populations of CD4^+^ and CD8^+^ T cells, both demonstrating poor expressions of interferon-γ and tumor necrosis factor-α. Both CD4^+^ and CD8^+^ effector T cells in transgenic mice displayed distinctive and differential expression of T-bet and Eomesodermin, respectively, in conjunction with high expression of programmed cell death protein-1 and other exhaustion-associated markers. Importantly, transgenic Aire expression did not result in any detectable changes in the population of Foxp3^+^ regulatory T (Treg) cells. Co-transfer experiments also demonstrated that Aire transgenic dendritic cells, as a “stand-alone” cell population, had the potential to suppress effector T cells *in vivo* without the support of Treg cells, but eventually failed to prevent the diabetogenesis in recipient mice. In conclusion, our study suggests that apart from its role in clonal deletion of autoreactive T cells or clonal diversion to Treg lineage, Aire can also contribute to tolerance by forcing effector T cells into a state of exhaustion with poor effector functions, thereby effectively containing autoimmune diseases.

## Introduction

Type 1 diabetes (T1D) is an autoimmune disease with a very early onset that manifests in genetically susceptible children at less than 10 years of age (1). The epidemiology of T1D shows an uneven global distribution with varying incidences reported from different regions (2). It is primarily a T helper 1 cell-mediated autoimmune disease in which autoreactive T cells selectively destroy pancreatic β cells resulting in insufficient insulin production (3).

Inefficient central tolerance is one of the primary causative factors underlying the pathogenesis of T1D, allowing the selection, and escape of self-reactive T cells from the thymus. Among these self-reactive T cells are those that target pancreatic auto-antigens such as insulin (4). Autoimmune regulator (*Aire*) is an essential component of the process of negative selection that eliminates potential self-reactive T cells from the thymus. Aire is a transcriptional regulator primarily expressed in medullary thymic epithelial cells (mTECs) in the thymus (5). It is responsible for promiscuous expression of a number of peripheral antigens in the mTECs, facilitating their expression, and presentation to cognate thymocytes, failing which may lead to escape of self-reactive T cells from the thymus (6). Mutations in the *Aire* gene lead to the development of autoimmune polyendocrinopathy candidiasis ectodermal dystrophy, a monogenic disorder characterized by pervasive autoimmune manifestations such as hypoparathyroidism, ovarian failure, T1D, and alopecia (7). Inactivation of *Aire* in mice leads to autoimmune manifestations affecting various organs, although the organs targeted and the severity of lymphocytic infiltration are strongly correlated with the genetic background of mice studied (8, 9). Apart from the well-defined role of Aire-expressing mTECs in deletion of self-reactive thymocytes during negative selection (10, 11); Aire has also been reported to be involved in selection of Foxp3^+^ regulatory T (Treg) cells in the thymus (12, 13). It is now understood that Aire does not simply drive a given thymocyte toward deletion during negative selection, but can also “divert” it toward the Treg lineage (14). Thus, it can be argued that Aire is a crucial regulator of both “clonal deletion” and “clonal diversion” of a given thymocyte. Moreover, thymic Aire expression can be affected by female sex hormones such as estrogen and progesterone, which may explain why females are at higher risk of developing autoimmune diseases than males in both mice and humans (15).

Apart from thymic mTECs, Aire-expressing cells have also been identified in the peripheral lymphoid organs. These cells are phenotypically reminiscent of conventional antigen-presenting cells and, like mTECs, are capable of expressing several tissue-specific antigens (TSAs). Although there is little overlap between the TSAs expressed by mTECs and those expressed by peripheral Aire-expressing cells, these peripheral cells are still capable of presenting antigens to cognate T cells, leading to their deletion (16). Although the existence of Aire-expressing cells in the periphery suggests that such cells could contribute to peripheral tolerance, potentially complementing the shortcomings in central tolerance, their identity, and possible mechanism of tolerance imposed by these cells requires further investigation.

Here, we report that transgenic expression of *Aire* under control of a dendritic cell (DC)-specific promoter significantly attenuates autoimmune diabetes in non-obese diabetic (NOD) mice. DC-specific Aire expression in transgenic mice pushes CD4^+^ and CD8^+^ effector T cells into a state of “exhaustion.” This affects the expression of pro-inflammatory cytokines interferon-γ (IFN-γ) and tumor necrosis factor-α (TNF-α) which are intimately associated with the pathogenesis and exacerbation of autoimmune diabetes. Exhausted CD4^+^ and CD8^+^ T cells in transgenic mice are governed by unique transcriptional programs and display signature markers associated with exhaustion such as CD272 and CD160. In addition, tolerance induced in both CD4^+^ and CD8^+^ T cell subsets in transgenic mice appears to be largely antigen-specific rather than generalized in nature. A delayed onset of diabetes in recipient mice after adoptive transfer of splenocytes from transgenic mice suggests that transgenic DCs have tolerogenic properties. However, a limited protective efficacy of DC-T cell co-transfer experiment suggests that Aire transgenic DCs as a stand-alone population may require “help” from bystander lymphocyte populations.

## Materials and Methods

### Mice

NOD/Sytwu (K^d^, D^b^, I-A^g7^, I-E^null^), NOD-Rag1^−/−^, and NOD-BDC2.5 TCR transgenic mice were procured from The Jackson Laboratory (Bar Harbor, ME, USA). NOD-SCID mice were purchased from National Laboratory Animal Center (Taipei, Taiwan). All the mice were subsequently housed in specific pathogen-free facility provided by the animal center of National Defense Medical Center (Taipei, Taiwan). Experimental protocols requiring the use of mice were approved by the Institutional Animal Care and Use Committee of National Defense Medical Center.

### Generation of pCD11c-Aire Transgenic Mice

Autoimmune regulator cDNA was cloned from NOD mouse thymus and inserted into pBlueScript-II vector by Acc651 and XbaI double digestion, followed by ligation. Aire cDNA was linearized and blunt ended using DNA Polymerase I Large (Klenow) Fragment. Resulting construct was ligated downstream of CD11c promoter. pCD11c-Aire construct was spliced out using NotI–SalI double digestion for pronuclei microinjection.

### Analysis of Spontaneous Diabetes and Insulitis

To evaluate spontaneous diabetes frequency, urine glucose of female transgenic and littermate control mice was measured twice a week using Chemstrips (Boehringer Mannheim, Indianapolis, IN, USA). Mice with a urine glucose concentration >500 mg/dl in two consecutive tests were considered diabetic. For insulitis assessment, pancreases were collected from 12- to 15-week-old mice and fixed in 10% buffered formalin. Hematoxylin- and eosin-stained pancreas sections were blindly scored as described previously (17).

### Flow Cytometric Analysis

For phenotypic analysis of DCs, splenic lymphocyte suspensions were stained with fluorochrome conjugated antibodies specific for the following surface markers: CD11c (N418), CD80 (16–10 A1), CD86 (GL1), CD40 (39164), MHCII (10.2.16), and PDL1 (10F.9G2), all of which were purchased from eBioscience (eBioscience, San Diego, CA, USA). Cell suspensions of thymus, spleen, and pancreatic lymph nodes (PLNs) were stained and analyzed with antibodies against CD4 (RM4-5), CD8α (53-6.7), CD25 (PC61.5), Foxp3 (FJK-16s), IFN-γ (XMG1.2), IL-4 (11B11), IL-2 (JES6-5H4), programmed cell death protein-1 (PD-1) (J43), T-bet (4B10), Eomesodermin (Eomes) (Dan11mag), CD160 (CNX46-3), CD244 (C9.1), CD272 (8F4), KLRG1, or killer cell lectin-like receptor G1 (2F1), all purchased from eBioscience. Antibodies against TNF-α (MP6-XT22), LAG3 or lymphocyte-activation gene 3 (C9B7W), and TIM3 or T cell immunoglobulin and mucin domain 3 (RMT3-23) were purchased from BioLegend (San Diego, CA, USA). For intracellular cytokine staining, splenocytes were stimulated with phorbol 12-myristate 13-acetate/PMA, ionomycin, and monensin (Sigma-Aldrich, St. Louis, MO, USA) for 6 h, followed by staining with antibodies. All analyses were performed on a FACSCalibur flow cytometer (BD Biosciences, San Jose, CA, USA) and analyzed using CellQuest software (BD Biosciences).

### Adoptive Transfer

Normoglycemic 12- to 14-week-old female NOD mice were used as donors to prepare splenocytes suspension. Female NOD-SCID recipient mice were injected with 2 × 10^7^ splenocytes via the retro-orbital plexus and checked twice a week for development of diabetes. For adoptive transfer to *Rag1*^−/−^ mice, effector T cells were isolated from 6- to 8-week-old normoglycemic female NOD mice using Pan T Cell Isolation Kit (Miltenyi Biotec, Bergisch Gladbach, Germany) and anti-mouse CD25 Monoclonal Antibody (eBioscience) according to the manufacturer’s instructions. For isolation of splenic DCs, CD11c-PE (N418; BioLegend) and Anti-R-PE Magnetic Particles–DM (BD Biosciences) were used according to the manufacturer’s instructions. T cells (7.5 × 10^6^) and DCs (7.5 × 10^5^) were mixed together and injected into *Rag1*^−/−^ mice via retro-orbital plexus. Mice were checked twice every week for diabetes or sacrificed 1 month after transfer for T cell analysis.

### Isolation of Pancreas Infiltrating Lymphocytes (PILs)

Pancreases were collected from 12- to 15-week-old female mice, digested with collagenase XI, and gradient purified using Histopaque 1077 (Sigma-Aldrich). Lymphocytes were collected from the interface layer and incubated with cell dissociation buffer (Life Technologies, Carlsbad, CA, USA). The resulting solution was passed through a cell strainer to prepare single cell suspension for flow cytometric analysis.

### Quantitative Real-time (RT)-PCR

Splenic DC RNA was isolated (Qiagen, Valencia, CA, USA) to prepare cDNA using SuperScript III synthesis kit (Invitrogen, Carlsbad, CA, USA) for PCR analysis. PCR was performed on a StepOnePlus Real-Time PCR System (Thermo Fisher, Bartlesville, OK, USA) using FastStart Universal SYBR Green Master Rox (Roche, Mannheim, Germany). Details of the target genes and primer sequences are provided in Table 1.

**Table 1:** Sequences of primers used in quantitative RT-PCR.

Genes	Forward primer (5′–3′)	Reverse primer (5′–3′)
*Aire*	ACCATGGCAGCTTCTGTCCAG	GCAGCAGGAGCATCTCCAGAG
*Gad67*	TGCAACCTCCTCGAACGCGG	CCAGGATCTGCTCCAGAGAC
*Fabp2*	AGACGGAACGGAGCTCAC	GCTCTTCAGCGTTGCTCC
*Chrna1*	GTGCTGGGCTCTTTCATCTC	TTCTGTGCGCGTTCTCATAC
*Tgn*	CTGTGGTGTTCTCAGCCTCA	TTGGCCTGAGTAGCAGAGGT
*Csna*	TGACTGGACCCTCCATTCTC	CCTTGATTCTCTCCGCTCAG
*Csng*	TCTGGCAAAGCACGAAATAAAGG	AGTTGTTTGGAAGAACACGCTA
*Ins2*	ACCTTCAGACCTTGGCACTG	GCTGGGTAGTGGTGGGTCTA
*IRBP*	AATGACTCGGTCAGCGAACTTT	CTGTCACACCACTGGTCAGGAT
*Mbp*	CCTCAGAGGACAGTGATGTGTTT	AGCCGAGGTCCCATTGTT
*Sp1*	TGCTCTTCTACTTGTCACCATGA	TGTTTGTCTCCGGGTCCT
*Chga*	CAGCAGCTCGTCCACTCTTT	GGACGCACTTCATCACCTTG
*Mog*	GTGTGCTGACTCTCATCG	CTCCAGGAAGACACAACC
*Rps29*	ACGGTCTGATCCGCAAATAC	AGCATGATCGGTTCCACTTG

### DC–T Cell Co-Culture

CD4^+^ effector T cells were isolated from transgenic and non-transgenic (non-Tg) mice using Mouse CD4 T Lymphocyte Enrichment Set–DM (BD Biosciences) and anti-mouse CD25 Monoclonal Antibody (eBioscience). T cells were cultured with equal numbers of splenic DCs from wild-type mice, in a 96-well plate. Co-culture was supplemented with proinsulin peptide (Table 2). After 48 h, 1μCi [methyl-^3^H] thymidine (PerkinElmer, Shelton, CT, USA) was added to each well and 16–18 h later [methyl-^3^H] thymidine incorporation was measured using a Packard TopCount Microplate Scintillation Counter.

**Table 2:** Peptide sequences used in this study.

Peptide	Sequence
Proinsulin	SHLVEALYLVCGERG
p31	YVRPLWVRME

### Statistical Analyses

The log-rank (Mantel–Cox) test was used for survival curve analysis of diabetes incidence. Student’s unpaired *t*-test was used for comparisons between different groups in other experiments. Data are shown as mean ± SEM.

## Results

### Generation of pCD11c-Aire Transgenic NOD Mice

**Figure 1:**
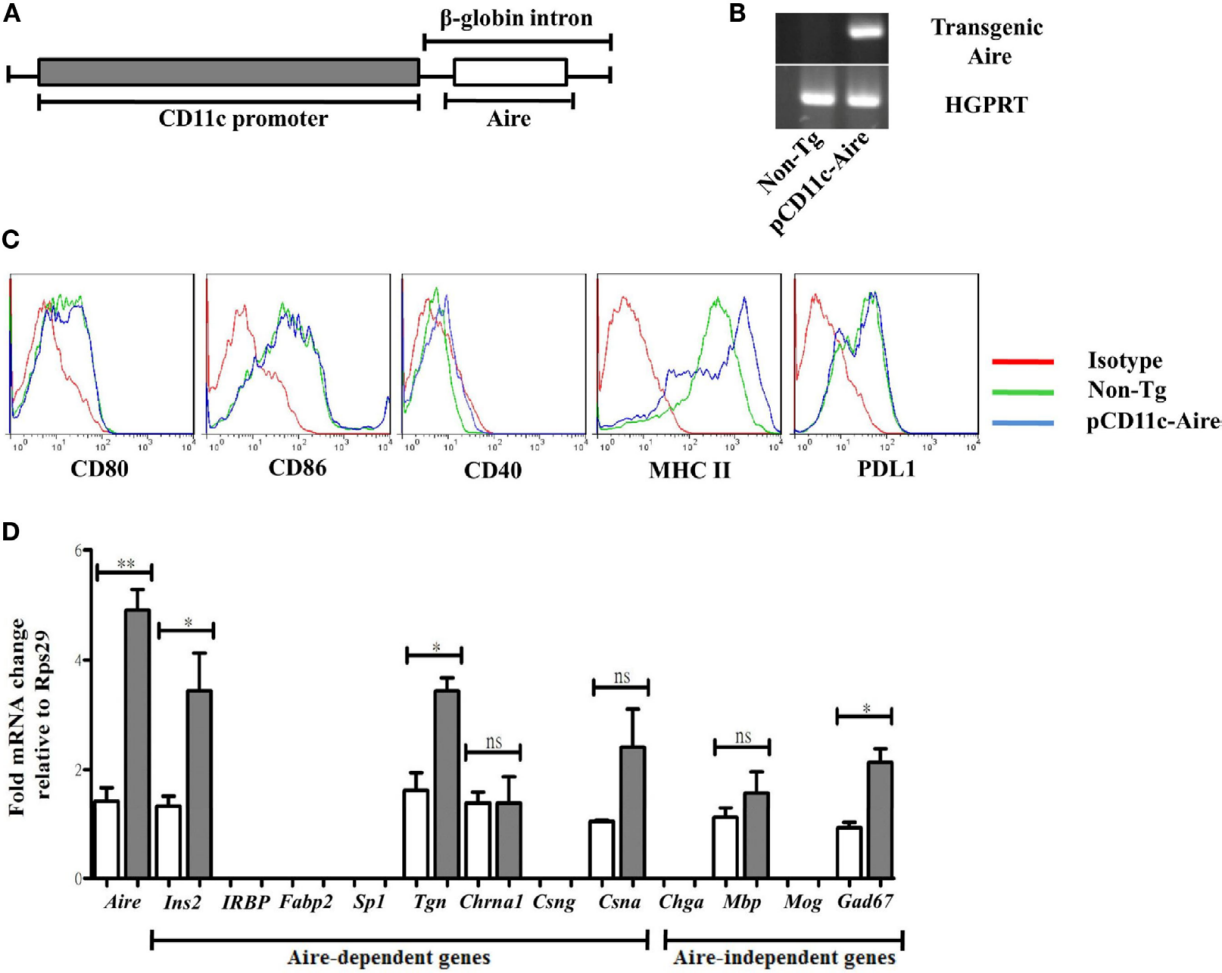
Generation of pCD11c-Aire transgenic non-obese diabetic mice. **(A)** Schematic diagram of pCD11c-Aire transgenic construct. Aire cDNA was cloned downstream of the CD11c promoter within a rabbit β-globin gene fragment that also contained an intron and a polyadenylation signal. **(B)** PCR genotyping of pCD11c-Aire and non-transgenic (non-Tg) littermate mice. Specifically designed primers were used to detect the transgenic Aire in genomic DNA (upper gel). HGPRT was used as internal control (lower gel). **(C)** Phenotypic characterization of splenic dendritic cells (DCs). Splenocytes were used for phenotypic analysis of splenic DCs using flow cytometry to analyze the surface expression of given cell surface markers. **(D)** Quantitative real-time (RT) PCR analysis of *Aire* and tissue-specific antigens. RNA was collected from splenic DCs and used as template for RT-PCR following cDNA synthesis, and normalized to *Rps29* (Ribosomal protein S29) mRNA expression. *IRBP*, interphotoreceptor retinoid binding protein; *Fabp2*, fatty acid-binding protein 2; *Sp1*, salivary protein-1; *Chrna1*, acetylcholine receptor alpha 1; *Csng*, casein γ; *Chga*, chromogranin A; *Mbp*, myelin basic protein; *Mog*, myelin oligodendrocyte glycoprotein; *Gad67*, glutamic acid decarboxylase. White bars represent non-Tg DCs, while gray bars represent transgenic DCs. Data are representative of at least three different experiments. *P*-values, ** = 0.0015, * ≤ 0.05, and ns = not significant.

Dendritic cells cross present antigens obtained not only *via* capture/endocytosis but also those synthesized by endogenous active transcription (18, 19). Because DCs play a crucial role in the pathogenesis of autoimmune diabetes, they are a suitable candidate for therapeutic intervention against this disease (20, 21). To study the role of Aire in DCs and their contribution to peripheral tolerance, we generated transgenic NOD mice that overexpress Aire under control of DC-specific CD11c/Itgax (integrin alpha X) promoter (Figure 1A), hereafter referred to as pCD11c-Aire mice. Transgenic mice could be reliably distinguished from non-Tg littermates by PCR genotyping using specific primers (Figure 1B). Phenotypic characterization of splenic DCs revealed that MHC II and CD40 were expressed at higher levels on transgenic DCs compared with their non-Tg counterparts (Figure 1C), suggesting that transgenic Aire-mediated enhanced expression of some surface molecules such as MHC II could potentially affect the immunogenic/tolerogenic properties of DCs.

One of the most crucial functions of Aire is to generate an imprint of peripheral TSAs in the thymus necessary for efficient screening of potential autoreactive T cells (6). As discussed above, this feature of Aire is not restricted to mTECs only, and peripheral Aire-expressing cells also express a number of TSAs that are for the most part distinct from those expressed by mTECs (16). To investigate whether DCs from pCD11c-Aire mice express TSAs and to determine their expression pattern, we examined a panel of TSAs consisting of Aire-dependent (6, 15, 22, 23) and Aire-independent (6, 23) target genes by quantitative RT-PCR. Our analyses revealed that some TSAs such as insulin 2 (*Ins2*) and thyroglobulin (*Tgn*) were expressed at significantly higher levels in transgenic DCs. Interestingly, Aire-independent *Gad67* exhibited significantly increased expression in transgenic DCs whereas some other target genes did not show any expression in either of the DC populations (Figure 1D). These observations suggest that DC-specific transgenic Aire expression in NOD mice can enhance expression of at least some of the Aire-dependent TSAs. In summary, we successfully established an “*in vivo*” model to investigate the potential role of DC-specific Aire in the maintenance of immune tolerance in NOD mice, and our data imply that transgenic Aire in DCs can modify their antigen presentation and/or stimulatory properties owing to enhanced expression of antigens and of crucial surface molecules.

### DC-Specific Transgenic Aire Expression Significantly Attenuates Autoimmune Diabetes in NOD Mice

**Figure 2:**
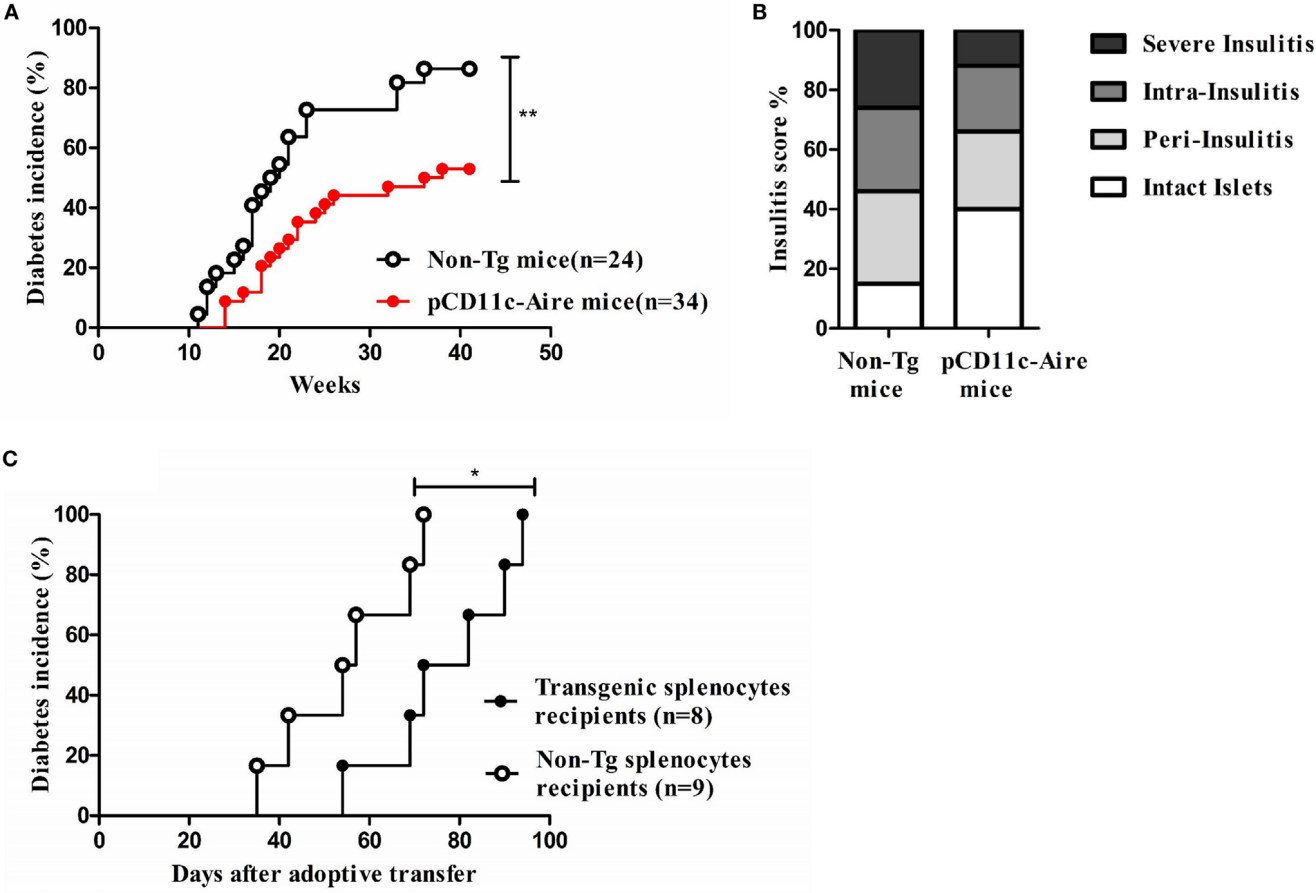
Dendritic cell-specific transgenic Aire expression significantly attenuates autoimmune diabetes in non-obese diabetic (NOD) mice. **(A)** Spontaneous diabetes incidence. Diabetes was detected by measuring urine glucose concentration for up to 40 weeks. Glycosuria was defined by urine glucose concentration >500 mg/dl in two consecutive tests. Open circles (*n* = 24) and solid red circles (*n* = 34) represent non-transgenic (non-Tg) and transgenic mice, respectively. *P*-value, ** = 0.0027 by log-rank test. **(B)** Assessment of insulitis. The severity of insulitis was evaluated in 10 pairs of 12- to 15-week-old transgenic and non-Tg mice by histological analysis of fixed pancreases. At least 100 islets were counted for each group. **(C)** Diabetes frequency following adoptive transfer of transgenic and non-Tg splenocytes. Splenocytes from non-Tg (open circles, *n* = 9 recipients) and transgenic mice (solid black circles, *n* = 8 recipients) were transferred to NOD-SCID recipients and diabetes was checked twice every week. *P*-value, * = 0.0279.

To assess the contribution of transgenic DCs toward peripheral tolerance and their possible effect on autoimmune diabetes, we evaluated the spontaneous diabetes incidence of transgenic and non-Tg female NOD mice. Transgenic NOD mice displayed moderately delayed onset of diabetes at 14 weeks of age compared with 11 weeks of age in non-Tg mice. With relatively slower disease kinetics among littermates, transgenic mice displayed significantly reduced incidence of spontaneous diabetes compared with their non-Tg littermates as monitored up to 40 weeks of age (Figure 2A). We also collected pancreases from 12- to 15-week-old transgenic and non-Tg mice for histological analysis of insulitis to corroborate the difference in disease severity. Significantly larger numbers of intact islets with minimal lymphocytic infiltration were observed in the pancreatic sections from transgenic mice. Also, there were significantly fewer islets showing overt immune infiltration in transgenic mice, suggesting that the diabetogenesis was attenuated compared with non-Tg mice (Figure 2B). To confirm possible reduction in diabetogenicity of the T cells of transgenic mice, we adoptively transferred splenocytes from transgenic and non-Tg mice into 8- to 10-week-old NOD-SCID recipients separately and followed up the onset of diabetes. Mice that received splenocytes from non-Tg mice developed diabetes much earlier than the mice that received splenocytes from transgenic mice (35 versus 54 days). In fact, 72 days after transfer, when all of the mice receiving splenocytes from non-Tg mice had developed diabetes, only 50% of the recipients of transgenic splenocytes had become diabetic (Figure 2C). These data suggest that transgenic Aire expression in DCs may attenuate the pathogenicity of effector T cells leading to significantly delayed onset and decreased severity of both spontaneous and lymphocyte transfer-induced diabetes.

### pCD11c-Aire Mice Have Reduced Populations of IFN-γ and TNF-α Expressing CD4^+^ and CD8^+^ Effector T Cells

**Figure 3:**
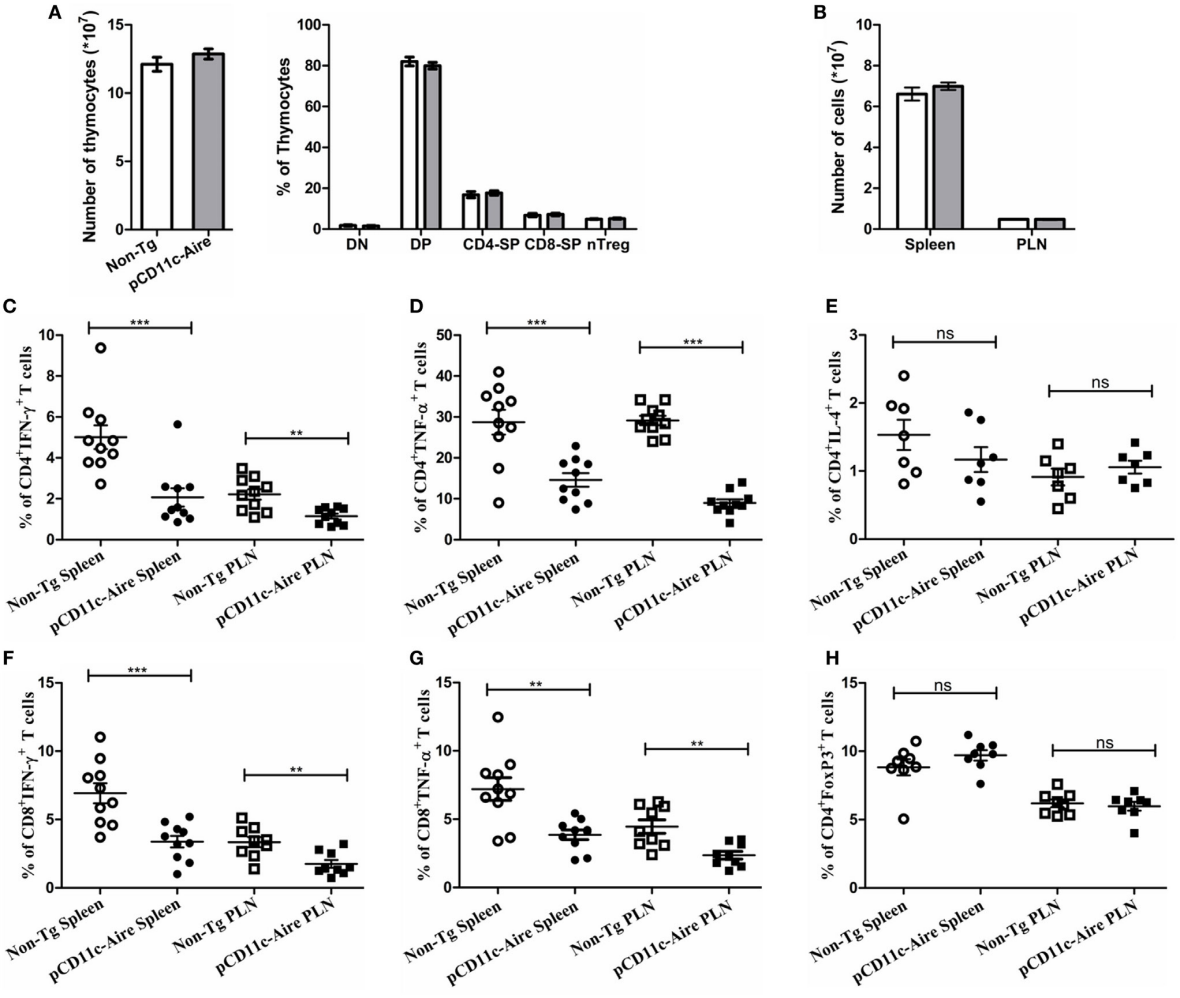
pCD11c-Aire mice have reduced populations of interferon-γ (IFN-γ) and tumor necrosis factor-α (TNF-α) expressing CD4^+^ and CD8^+^ effector T cells. **(A)** Thymocyte analysis. Thymocytes were collected from 8- to 10-week-old mice (*n* = 8 in each group) and analyzed for total cellularity and population of CD4^−^CD8^−^ (DN), CD4^+^CD8^+^ (DP), CD4^+^ (CD4-SP), CD8^+^ (CD8-SP), and CD4^+^FoxP3^+^ regulatory T cells. Non-transgenic and transgenic mice are represented by white and gray bars, respectively. **(B)** Spleen and pancreatic lymph nodes were collected from 8- to 10-week-old mice (*n* = 10 in each group) and analyzed for total cellularity. **(C,D)** IFN-γ and TNF-α expressing CD4^+^ T cell populations in the indicated organs. **(E)** IL-4 expressing CD4^+^ T cells in the indicated organs. **(F,G)** IFN-γ and TNF-α expressing CD8^+^ T cell populations in the indicated organs. **(H)** CD4^+^FoxP3^+^ regulatory T cell population in the indicated organs. *P*-values, ** ≤ 0.005, *** ≤ 0.0010, and ns = not significant.

Role of Aire in mTECs and the consequences of its deficiency are well documented. However, its role in DCs and subsequent effect on lymphocyte development/homeostasis is unclear. Thymocyte numbers and proportions of thymocyte subpopulations (DN, DP, CD4^+^, and CD8^+^) were indistinguishable between pCD11c-Aire transgenic and non-Tg mice. Notably, percentage of CD4^+^FoxP3^+^ natural regulatory T cells also remained unchanged in transgenic mice (Figure 3A). In the periphery, total cellularity of spleen and PLNs was largely similar in transgenic and non-Tg mice (Figure 3B). These results indicate that the protective phenotype mediated by DC-specific Aire is not likely to be mediated by the modulation of lymphocyte development in either central or peripheral lymphoid organs.

We further investigated whether cytokine expression by effector T cells was modulated in pCD11c-Aire mice. Indeed, we observed significant reduction in populations of IFN-γ and TNF-α expressing CD4^+^ T cells in both spleen and PLNs (Figures 3C,D, respectively), while IL-4 expressing CD4^+^ T cells remained virtually unchanged (Figure 3E), suggesting that amelioration of diabetes in transgenic mice may not be due to overriding effect of IL-4 expressing T helper 2 cells. Interestingly, we also observed significant reductions in the populations of IFN-γ and TNF-α expressing CD8^+^ T cells in spleen and PLNs of transgenic mice, with a decrease in magnitude similar to CD4^+^ T cells (Figures 3F,G, respectively). These data suggest that reduced expression of pro-inflammatory cytokines IFN-γ and TNF-α in both CD4^+^ and CD8^+^ T cells is responsible for the attenuation of autoimmune diabetes in transgenic mice.

We also examined the potential variation in peripheral Treg cells in transgenic mice. Similar to our observations in thymus (Figure 3A), the data did not show any significant changes in Treg cells in spleen or PLNs (Figure 3H). Overall, these findings suggest that attenuation of autoimmune diabetes in pCD11c-Aire mice was largely due to the overall reduction in IFN-γ and TNF-α secreting T cells in both CD4^+^ and CD8^+^ subsets. These data could also explain the reduced diabetogenicity of splenocytes from pCD11c-Aire as demonstrated by adoptive transfer experiment (Figure 2C).

### pCD11c-Aire Mice Have Fewer and Less Pathogenic T Cells within the Pancreas

**Figure 4:**
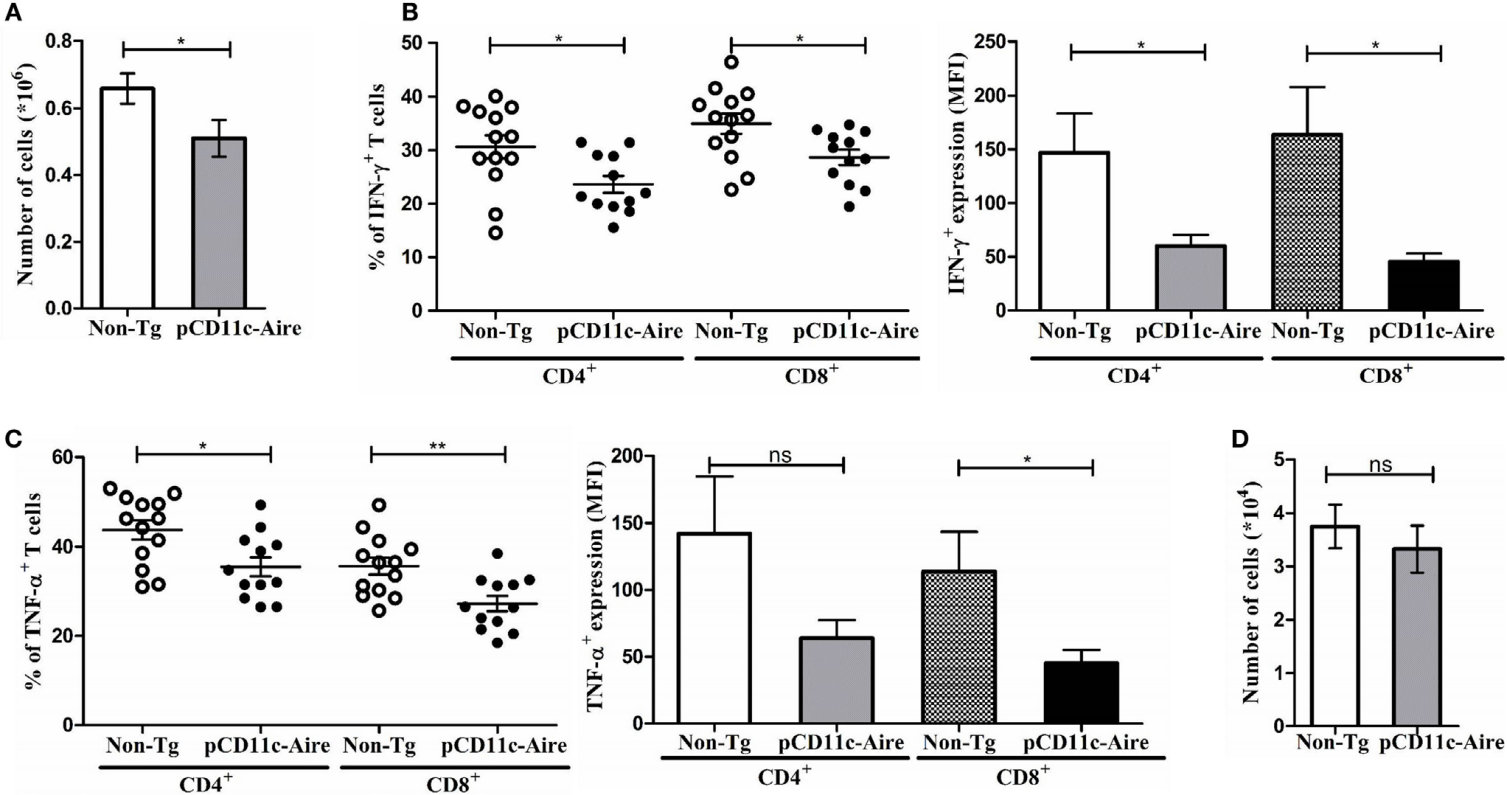
pCD11c-Aire mice have fewer and less pathogenic T cells within the pancreas. **(A)** Reduction in lymphocyte populations within transgenic mice pancreases. Pancreases were collected from 12- to 15-week-old non-transgenic (non-Tg) (*n* = 13) and transgenic (*n* = 12) mice, lymphocytes were isolated and enumerated. **(B)** Lymphocytes from pancreases were analyzed for interferon-γ (IFN-γ) expressing CD4^+^ and CD8^+^ T cells (left) and expression levels of IFN-γ (right). **(C)** Lymphocytes from pancreases were analyzed for tumor necrosis factor-α (TNF-α) expressing CD4^+^ and CD8^+^ T cells (left) and expression levels of TNF-α (right). **(D)** Population of regulatory T cells in non-Tg and transgenic mice pancreases. *P*-values, * ≤ 0.05, ** ≤ 0.0050, and ns = not significant.

Attenuation of diabetes in pCD11c-Aire mice was validated by histological analyses of pancreatic tissue sections which showed significantly reduced infiltration of lymphocytes into pancreatic islets (Figure 2B). We further expanded upon these findings by analyzing PILs from transgenic and non-Tg mice. There were significantly fewer lymphocytes recovered from the pancreases of transgenic mice (Figure 4A). In addition, as observed in spleen and PLN of transgenic mice (Figure 3), lymphocytes within the pancreases also exhibited significantly reduced population of IFN-γ secreting CD4^+^ and CD8^+^ T cells, coupled with noticeably lower levels of IFN-γ expression (Figure 4B). Similarly, there was a marked reduction in the populations of CD4^+^ and CD8^+^ T cells expressing TNF-α in pancreases of transgenic mice (Figure 4C). We further evaluated the possible accumulation of CD4^+^Foxp3^+^ Treg cells in pancreatic islets because they are crucial to subdue the effector T cells invading the pancreatic islets following an autoimmune breakdown (24). However, the population of CD4^+^Foxp3^+^ Treg cells within the pancreases was indistinguishable between transgenic and non-Tg mice (Figure 4D). We also observed a slight reduction in CD4^+^ T cells expressing IL-17 and IL-21 within the pancreases of transgenic mice, but this reduction was moderate compared with that of IFN-γ/TNF-α expressing CD4^+^/CD8^+^ T cells (data not shown). Together, these observations suggest that reduction in IFN-γ and TNF-α expressing pro-diabetogenic CD4^+^ and CD8^+^ T cells, without any enhanced contribution from Treg cells, likely accounts for attenuation of diabetes in pCD11c-Aire mice.

### pCD11c-Aire Mice Harbor “Exhausted” CD4^+^ and CD8^+^ T Cell Populations

**Figure 5:**
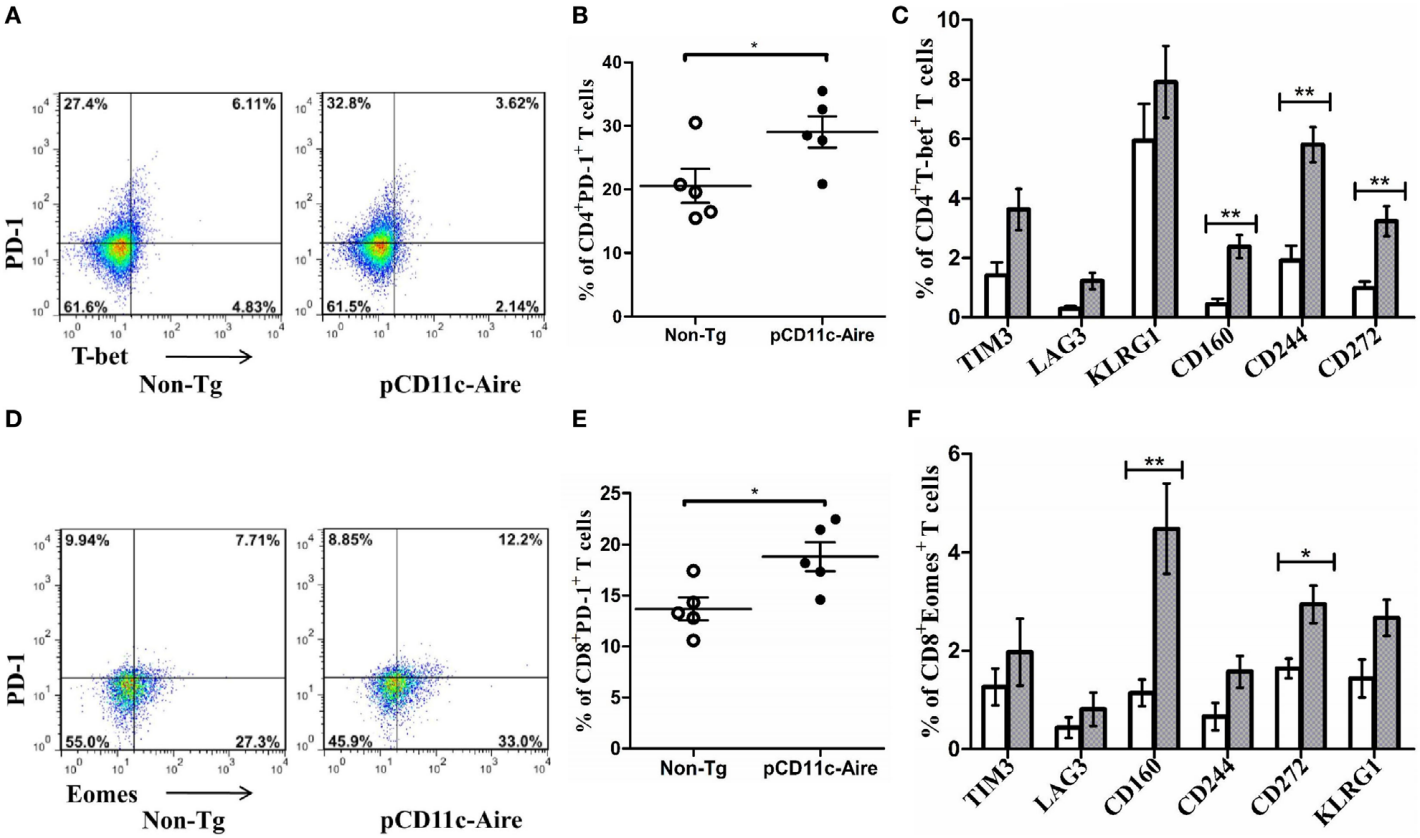
pCD11c-Aire mice harbor “exhausted” CD4^+^ and CD8^+^ T cell populations. **(A)** Programmed cell death protein-1 (PD-1) and T-bet expressing splenic CD4^+^ T cells in non-transgenic (non-Tg) and transgenic mice (*n* = 7 each). **(B)** PD-1 expression by splenic CD4^+^ T cells. **(C)** Population of splenic CD4^+^T-bet^+^ T cells with additional expression of the indicated markers associated with CD4^+^ T cell exhaustion. Non-Tg and transgenic mice are represented by white and gray bars, respectively. **(D)** PD-1 and eomesodermin (Eomes) expressing splenic CD8^+^ T cells in non-Tg and transgenic mice (*n* = 7 each). **(E)** PD-1 expression by splenic CD8^+^ T cells. **(F)** Population of splenic CD8^+^Eomes^+^ T cells expressing the indicated surface markers associated with CD8^+^ T cell exhaustion. *P*-values, * ≤ 0.05, ** ≤ 0.005.

Chronic exposure of T cells to cognate antigens may result in transition of T cells to a distinct phenotype akin to a state of exhaustion, which is characterized by higher expression of inhibitory receptors such as PD-1, LAG3, or TIM3 and reduced effector functions with lower expression of IFN-γ/TNF-α/IL-2 and cytotoxic molecules. Such “exhausted” T cells also have a distinct transcriptional profile characterized by the expression of transcription factors such as Bcl-6, Helios, Eomes, and basic leucine zipper ATF-like transcription factor (25, 26). We hypothesized that endogenous self-antigens induced by transgenic Aire in DCs may be robustly presented to cognate T cells in the context of stronger MHCII expression and co-stimulatory signals, which during a course of persistent stimulation may push T cells into “exhaustion.” Having confirmed that transgenic mice had significantly reduced populations of IFN-γ and TNF-α expressing CD4^+^ T cells (Figures 3C,D), we next analyzed CD4^+^ T cells for signs of exhaustion. Our results demonstrated that CD4^+^ T cells from transgenic mice exhibited lower expression of transcription factor T-bet coupled with reciprocally higher expression of inhibitory receptor PD-1 (Figures 5A,B), which is characteristic of an “exhausted” CD4^+^ T cell phenotype (27). We also examined the expression of other surface markers typically associated with CD4^+^ T cell exhaustion such as TIM3, LAG3, CD160, KLRG1, CD244 (2B4), CD272 (24, 25) and found significantly higher expression of CD160, CD244, and CD272 (Figure 5C) in CD4^+^ T cells from transgenic mice. These observations indicate that the CD4^+^ T cells in transgenic mice are in a state of relative exhaustion suggestive of suppressed pathogenicity of CD4^+^ T cells.

We also analyzed CD8^+^ T cells from the perspective of exhaustion. Various transcription factors have been associated with CD8^+^ T cell exhaustion such as Eomes, T-bet, and Blimp-1. CD8^+^ T cells from transgenic mice displayed a significantly larger population of “exhausted” cells with simultaneously higher expression of Eomes and PD-1, compared with CD8^+^ T cells from non-Tg mice (Figures 5D,E). Examining additional exhaustion-associated markers in CD8^+^ T cells from transgenic mice, we observed markedly higher expression of CD160 and CD272, compared with CD8^+^ T cells from non-Tg mice (Figure 5F). Together, these observations suggest that both CD4^+^ and CD8^+^ T cells in pCD11c-Aire mice are in a state of “exhaustion” causing them lose their effector functions and becoming of a less pathogenic effector T cells.

### Tolerance Induction by CD11c-Aire DCs Is Largely Auto-Antigen Specific

**Figure 6:**
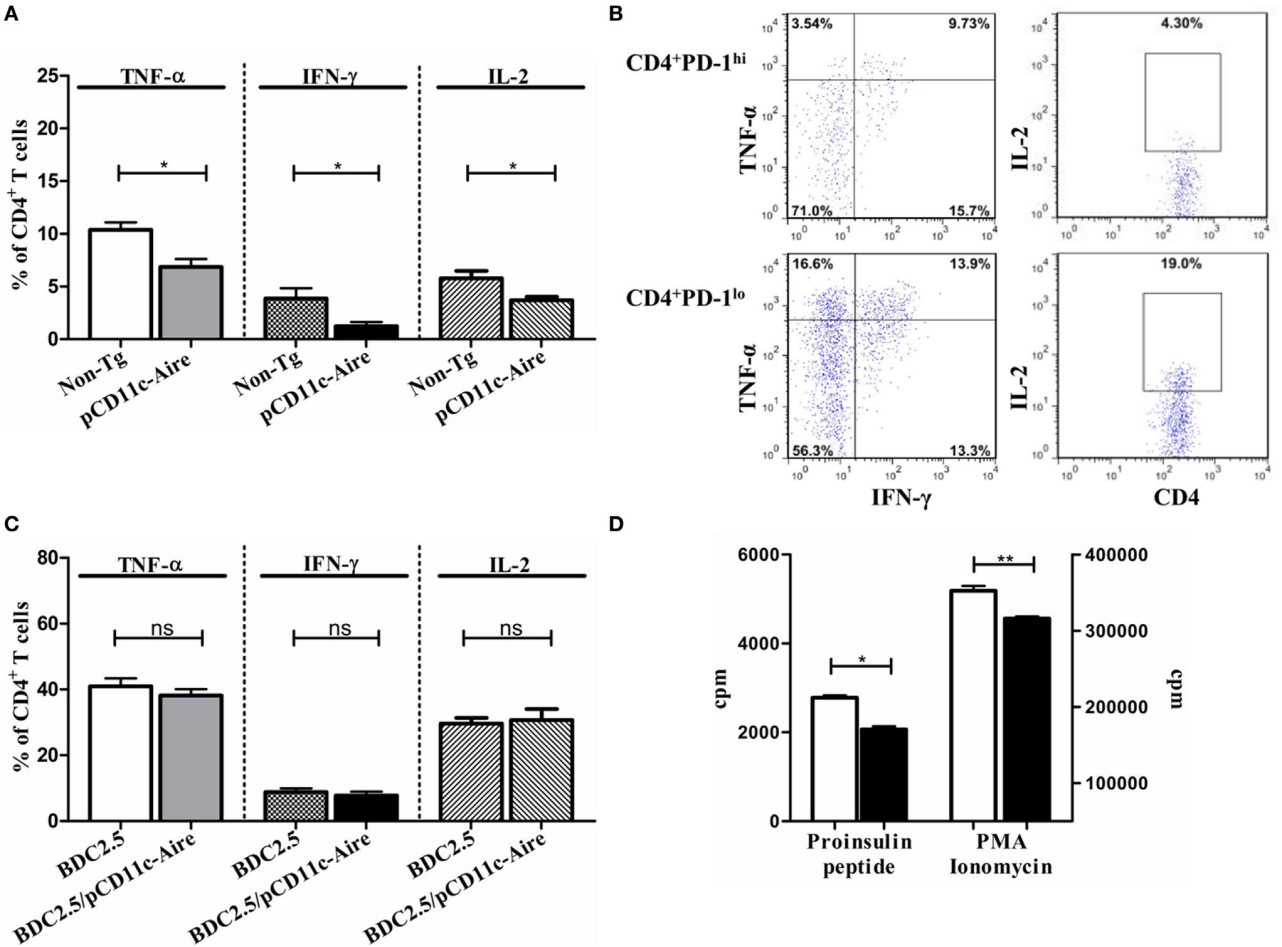
Tolerance induction by pCD11c-Aire dendritic cells (DCs) is largely auto-antigen specific. **(A)** Effector cytokine expression by proinsulin-specific splenic CD4^+^ T cells. Splenocytes from non-transgenic (non-Tg) and transgenic mice were stimulated with proinsulin peptide for 18–24 h before analysis. **(B)** Effector cytokine expression by proinsulin peptide stimulated splenic CD4^+^ programmed cell death protein-1 (PD-1)^lo^ and CD4^+^PD-1^hi^ T cells in transgenic mice. **(C)** Splenic CD4^+^ T cell effector cytokine expression in BDC2.5 and BDC2.5/pCD11c-Aire mice. Splenocytes from BDC2.5 and BDC2.5/pCD11c-Aire mice were stimulated with p31 mimotope peptide (Table 2) for 18–24 h before analysis. **(D)** Splenic CD4^+^ T_eff_ cell proliferation. Non-Tg or transgenic mice splenic CD4^+^ T cells were cultured with non-Tg mice splenic DCs in the presence of proinsulin peptide for 3 days before [methyl-^3^H] thymidine incorporation was measured (left). In addition, indicated CD4^+^T cells were stimulated non-specifically using PMA and ionomycin (right). White bars represent non-Tg mice CD4^+^ T cell proliferation whereas black bars represent transgenic mice CD4^+^ T cell proliferation. *P*-values, * ≤ 0.05, ** = 0.0062, and ns = not significant.

Although our findings *prima facie* suggest that T cell exhaustion is behind the attenuation of autoimmune diabetes in transgenic mice, we cannot rule out the possibility of a broadly acting comprehensive mode of tolerance. Because our analysis showed that transgenic DCs express significantly higher levels of *Ins2* auto-antigen than non-Tg DCs (Figure 1D), we hypothesized that enhanced *Ins2* expression and presentation by transgenic DCs might push insulin-reactive T cells into exhaustion, subduing their effector properties in the process. To examine the effector cytokine expression by insulin-reactive CD4^+^ T cells in transgenic mice, we stimulated their splenic CD4^+^ T cells *in vitro* with an immunodominant proinsulin peptide (28). Our results revealed that CD4^+^ T cells in transgenic mice had significantly reduced expression of TNF-α, IFN-γ, and IL-2 after they were stimulated with proinsulin peptide (Figure 6A), compared with CD4^+^ T cells from non-Tg mice. In addition, using PD-1 as a surrogate marker for exhausted CD4^+^ T cells in transgenic mice, we found that CD4^+^PD-1^hi^ T cells expressed significantly less TNF-α and IL-2 compared with CD4^+^PD-1^lo^ T cells. Unexpectedly, IFN-γ was expressed at marginally higher levels in CD4^+^PD-1^hi^ T cells (Figure 6B). These results suggest that enhanced expression and presentation of TSAs (in this case *Ins2*) by transgenic DCs potentially enforces the exhaustion of cognate effector T cells. Thus, it can be argued that transgenic DCs elicit tolerance predominantly in an antigen-specific manner *in vivo*.

To confirm our finding that transgenic DCs induce largely antigen-specific tolerance in an Aire-dependent manner, we turned to BDC2.5 TCR transgenic mice which contain a rearranged transgenic CD4^+^ T cell receptor that recognizes the antigenic peptide derived from Chromogranin A presented in the context of MHCII haplotype I-A^g7^ (29, 30). We cross-bred pCD11c-Aire mice with BDC2.5 transgenic mice to generate BDC2.5/pCD11c-Aire double transgenic mice. As per our analysis, neither transgenic nor non-Tg DCs express *Chromogranin A* (Figure 1D). We contended that since transgenic DCs were devoid of *Chromogranin A* expression, they should not be able to tolerize CD4^+^ T cells of BDC2.5/pCD11c-Aire mice in an antigen-specific manner. We indeed observed no significant differences in TNF-α, IFN-γ, and IL-2 expression by CD4^+^ T cells of BDC2.5 or BDC2.5/pCD11c-Aire mice (Figure 6C). In fact, in some instances, effector cytokine expression was even higher in BDC2.5/pCD11c-Aire mice, suggesting that CD4^+^ T cells were not necessarily in a tolerized state. These observations support our argument that ectopic expression of an auto-antigen by transgenic DCs enables them to suppress its cognate effector T cells *in vivo*.

Reduction/loss of proliferative potential is one of the hallmark features of T cells undergoing exhaustion. We sought to determine whether antigen-specific T cells with poor effector cytokine expression also had reduced proliferative potential. For this, we set up a DC-T cell co-culture involving non-Tg DCs loaded with proinsulin peptide and CD4^+^ effector T cells from non-Tg or transgenic mice. We found that CD4^+^ T cells from transgenic mice proliferated much poorly in response to proinsulin peptide, than their non-Tg counterparts (Figure 6D). This suggests that insulin-reactive CD4^+^ T cells in transgenic mice have poor proliferative potential resulting from antigen-induced exhaustion and therefore respond poorly to cognate antigen *in vitro*. CD4^+^ effector T cells from transgenic mice again showed significantly reduced proliferation compared with CD4^+^ effector T cells from non-Tg mice, when they were stimulated non-specifically by PMA and ionomycin (Figure 6D). This observation further supports our contention that CD4^+^ effector T cells from transgenic mice are in a state of exhaustion. Overall, the presence of the features characteristic of exhausted T cells indicates that the tolerance induced by Aire in transgenic mice is largely auto-antigen specific.

### DC–T Cell Co-Transfer Experiments Reveal Transgenic DCs to Have Strong Tolerogenic But Limited Disease Preventive Potential

**Figure 7:**
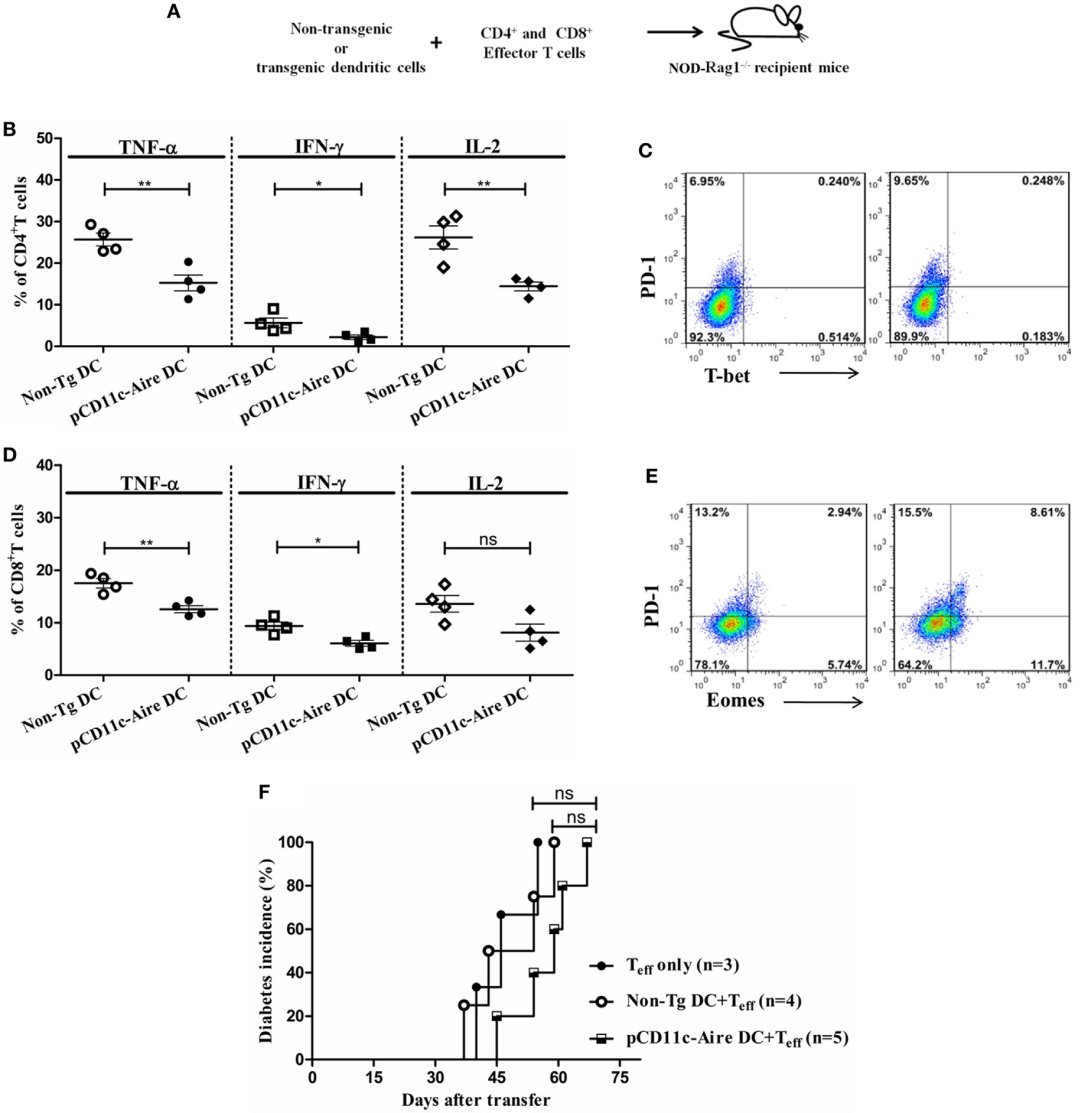
Dendritic cell (DC)–T cell co-transfer experiments reveal transgenic DCs to have strong tolerogenic but limited disease preventive potential. **(A)** Schematic diagram of DC-T cell co-transfer into *RAG1*^−^*^/^*^−^ mice. DCs from non-transgenic (non-Tg) or transgenic mice were mixed with CD4^+^ and CD8^+^ effector T cells and transferred into recipient mice for diabetes incidence and T cell analysis. **(B)** CD4^+^ T cell effector cytokine expression of mice recipient with non-Tg or transgenic DCs and effector T cells. **(C)** T-bet and programmed cell death protein-1 (PD-1) expression in CD4^+^ T cells from non-Tg DC (left) and transgenic DC (right) recipient *RAG1*^−^*^/^*^−^ mice. **(D)** CD8^+^ T cell effector cytokine expression of mice recipient with non-Tg or transgenic DCs and effector T cells. **(E)** Eomesodermin and PD-1 expression in CD8^+^ T cells from non-Tg DC (left) and transgenic DC (right) recipient *RAG1*^−^*^/^*^−^ mice. **(F)** Diabetes incidence in *RAG1*^−^*^/^*^−^ mice recipient with effector T cells with/without indicated DC populations. *P*-values, * ≤ 0.05, ** ≤ 0.0010, and ns = not significant.

To assess the tolerogenic potential of Aire transgenic DCs as a stand-alone population, we adoptively transferred DCs collected from non-Tg or transgenic mice together with CD4^+^ and CD8^+^ effector T cells from wild-type NOD mice into *Rag1*^−/−^ immunodeficient mice and analyzed T cell 30 days after transfer (Figure 7A). Analysis of CD4^+^ T cells recovered from the recipients of transgenic DCs revealed significantly reduced populations of TNF-α, IFN-γ, and IL-2 expressing cells compared with CD4^+^ T cells from non-Tg DC recipients (Figure 7B). Moreover, the percentage of CD4^+^ T cells with higher PD-1 but lower T-bet expression was much larger in mice that received transgenic DCs, further indicating that there was a larger population of exhausted T cells in these recipients (Figure 7C). These *in vivo* observations also suggest that a limited number of transgenic DCs have the potential to render the CD4^+^ T cells into exhaustion, with attenuated expression of effector cytokines.

Similarly, CD8^+^ T cells collected from recipients of transgenic DCs had significantly reduced populations of TNF-α and IFN-γ expressing cells, although IL-2 expressing population was indistinguishable from non-Tg DC recipients (Figure 7D). In addition, analysis of Eomes and PD-1 expression revealed that there was a larger population of Eomes^+^PD-1^+^ cells within transgenic DCs recipients (Figure 7E). Collectively, our results suggest that a stand-alone population of transgenic DCs has strong potential to suppress the CD4^+^ and CD8^+^ effector T cells by means of enforcing the exhaustion of these cells. These results also suggest that transgenic DCs could modulate these effects without any support from bystander B or T lymphocytes such as Treg cells, in *Rag1*^−/−^ immunodeficient mice.

To further evaluate whether the tolerogenic potential of these stand-alone DCs could also be extended to diabetes suppression, we carried out similar DC-T cell co-transfer experiment and examined the recipient mice for development of diabetes. As control group, we included *Rag1*^−/−^ mice that received only effector T cells. Mice transferred with effector T cells alone or effector T cells plus non-Tg DCs showed similar diabetic onset at day 40 and day 37 after transfer, respectively (Figure 7F). However, recipients of effector T cells and transgenic DCs showed a slightly delayed onset at 45 days after transfer (Figure 7F). Beyond the time of onset, the transgenic DCs appeared to have a suppressive effect on disease kinetics, although this was not as significant. Overall, these results suggest that although a stand-alone population of Aire transgenic DCs *in vivo* has stronger tolerogenic potential against effector T cells, they fail to reflect this tolerogenic effect in terms of protection from autoimmune diabetes. It is likely that this population requires some “help” from other bystander lymphoid cells, without which DCs may not be capable of maintaining an immunosuppressive environment.

## Discussion

Non-obese diabetic mouse thymus undergoes age-associated progressive involution that can affect thymocyte selection (31–33). This is exacerbated by a flawed effector and Treg cell selection process, expediting the prospects of autoimmune pathogenesis (34, 35). Progressive thymic involution is accompanied by a gradual decrease in Aire expression by mTECs, leading to the reduction in the expression of Aire-dependent TSAs (15, 36). Apart from the thymic involution and a “leaky” central tolerance, autoimmune diabetes in NOD mice is also worsened by shortcomings in peripheral tolerance that influence both effector and regulatory T cell compartments (37). Therefore, it is appropriate to consider the tolerogenic potential and contribution of extrathymic Aire-expressing cells against the pathogenesis of autoimmune diseases, especially in the event of thymic Aire deficiency.

T cell exhaustion is a commonly occurring phenomenon during chronic infections and cancer development. Microbial infections and cancerous tissues, by means of T cell exhaustion, advantageously escape the host immune system, allowing them to persist for extended periods. For this reason, novel strategies are being worked on to reverse the T cell exhaustion so that the host immune system can mount an effective response against cancers or infections (38, 39). However, a recent study suggests that the transcriptional profile of exhausted CD8^+^ T cells during chronic infections may actually predict favorable clinical outcome in autoimmune and inflammatory diseases (40). Therefore, exhaustion may on one hand promote chronic infection/cancer, but on the other hand may actually alleviate autoimmune diseases. Tolerogenic potential of pCD11c-Aire DCs is also in agreement with previously reported extrathymic Aire-expressing cells that were significantly capable of preventing autoimmune diabetes by inducing functional inactivation or deletion of effector T cells in secondary lymphoid organs (16, 41). Thus, our study further reinforces extrathymic role of Aire-expressing cells in the periphery and their contribution in peripheral tolerance.

Dendritic cell–T cell co-transfer experiments helped us properly evaluate the tolerogenic potential of stand-alone DC population. Our findings suggest that a limited number of transgenic DCs are capable of pushing effector CD4^+^/CD8^+^ T cells into a state of exhaustion. Adoptively transferred DCs could suppress the diabetes in recipient mice in a substantial manner, although transgenic mice had much significant relief from autoimmune diabetes. There could be several explanations for this observation. First, *Rag1*^−/−^ recipient mice had their own residential DC population and it is possible that these residential DCs may dampen the suppressive environment provided by adoptively transferred transgenic DCs in terms of diabetic suppression. In addition, transgenic mice were privileged with a tolerogenic environment provided by Aire transgenic DCs from birth, helping in efficient suppression/attenuation of autoimmune diabetes. Second, we transferred T cells devoid of Tregs into recipient mice. The role of Tregs in prevention/delay of autoimmune diabetes in NOD mice is undisputed and there is a possibility that transgenic DCs require some contribution from bystander Treg cells in order to prevent the diabetes onset. Finally, it is simply possible that number of DCs transferred was just not large enough to prevent diabetes in the recipient mice, especially when T cells underwent proliferation and expanded in numbers beyond the control of DCs.

Autoimmune regulator is involved in T cell development in the thymus and lack of Aire expression can disrupt both effector and Treg cell repertoires. Aire is responsible for the fate determination of developing thymocytes and acts as a switch to the “clonal diversion” of thymocytes toward distinct lineages (12–14). Our study reveals that DC-specific expression of Aire does not cause any significant changes in Treg population of transgenic mice, suggesting that Treg cells may not be primarily responsible for the attenuation of autoimmune diabetes in transgenic mice. However, the possibility of fluctuations in Treg cell pool at specific clonotype level cannot be entirely ruled out. It is likely that transgenic Aire expression may induce subtle changes, qualitative if not quantitative, in Treg cell pool which may contribute to the protection from autoimmune manifestations. This factor may also be relevant for T cells isolated from the pancreases of transgenic mice where population of Treg cells was although similar to non-Tg mice pancreases, yet degree of insulitis was much less severe. Therefore, whether DC-specific transgenic Aire expression has the potential to induce qualitative and/or quantitative changes in Treg cell pool; changes that are capable of containing the autoimmune phenotype, requires further investigation.

In summary, our study elucidates a positive role of Aire in peripheral tolerance and illustrates the relative contribution of Aire in combination with different lymphocyte subsets in protection from autoimmune diabetes. Our study is also the first to suggest T cell exhaustion as a mechanism employed by Aire-expressing cells to attenuate effector T cells, thereby suppressing autoimmune diabetes in NOD mice. This T cell exhaustion encompassed both CD4^+^ and CD8^+^ T cell populations. Adoptive transfer experiments helped establish transgenic DCs as a stand-alone cell population that is capable of rendering the effector T cells into a state of exhaustion. However, the diabetic outcome in the adoptive transfer experiments suggests that these DCs may require “help” from bystander lymphocyte populations. The findings of our study hold substantial therapeutic potential and could be used to design novel antigen-specific immunomodulatory approaches (42, 43), in preventing and/or treating autoimmune diseases.

## Ethics Statement

This study was carried out in accordance with the recommendations of Institutional Animal Care and Use Committee of National Defense Medical Center. The protocol was approved by the Institutional Animal Care and Use Committee.

## Author Contributions

DK and H-KS conceptualized and planned the study. DK, L-TY, M-WC, and F-CC performed the experiments and analyzed the data. DK and H-KS wrote the manuscript and H-KS supervised the study.

## Conflict of Interest Statement

The authors declare that the research was conducted in the absence of any commercial or financial relationships that could be construed as a potential conflict of interest. The reviewer MW and handling Editor declared their shared affiliation.
